# Challenges to implementing Gavi’s health system strengthening support in Chad and Cameroon: results from a mixed-methods evaluation

**DOI:** 10.1186/s12992-017-0310-0

**Published:** 2017-11-16

**Authors:** Emily Dansereau, Yodé Miangotar, Ellen Squires, Honoré Mimche, UND group, Alexis Ngarmbatedjimal, Haroun Koumakoi, Djimet Seli, IFORD group, Hénoque Blaise Nguendo Yongsi, Julien Guy Ewos Bomba, Léopold Cyriaque Donfack Mbasso, Mariane Kenmegni Omgba, Patrice Tanang Tchouala, Vivien Meli Meli, Charbel El Bcheraoui

**Affiliations:** 1 Institute for Health Metrics and Evaluation, University of Washington, 2301 5th Avenue, Suite 600, Seattle, WA 98121 USA; 2 University of N’Djamena, Avenue Mobutu, BP 1117 N’Djamena, Chad; 3 Institut de Formation et de Recherche Démographiques, University of Yaoundé II, 1556 Yaoundé, Cameroon

**Keywords:** Health system strengthening, HSS, Gavi, Implementation science, Process evaluation, Chad, Cameroon

## Abstract

**Background:**

Since 2005, Gavi has provided health system strengthening (HSS) grants to address bottlenecks affecting immunization services. This study is the first to evaluate the Gavi HSS implementation process in either Cameroon or Chad, two countries with significant health system challenges and poor achievement on the child and maternal health Millennium Development Goals.

**Methods:**

We triangulated quantitative and qualitative data including financial records, document review, field visit questionnaires, and key informant interviews (KII) with representatives from the Ministries of Health, Gavi, and other partners. We conducted a Root Cause Analysis of key implementation challenges, guided by the Consolidated Framework for Implementation Research.

**Results:**

We conducted 124 field visits and 43 KIIs in Cameroon, and 57 field visits and 39 KIIs in Chad. Cameroon’s and Chad’s HSS programs were characterized by delayed disbursements, significant deviations from approved expenditures, and reprogramming of funds. Nearly a year after the programs were intended to be complete, many district and facility-level activities were only partially implemented and significant funds remained unabsorbed. Root causes of these challenges included unpredictable Gavi processes and disbursements, poor communication between the countries and Gavi, insufficient country planning without adequate technical assistance, lack of country staff and leadership, and weak country systems to manage finances and promote institutional memory.

**Conclusions:**

Though Chad and Cameroon both critically needed support to strengthen their weak health systems, serious challenges drastically limited implementation of their Gavi HSS programs. Implementation of future HSS programs in these and similar settings can be improved by transparent and reliable procedures and communication from Gavi, proposals that account for countries’ programmatic capacity and the potential for delayed disbursements, implementation practices that foster learning and adaptation, and an early emphasis on developing managerial and other human resources.

**Electronic supplementary material:**

The online version of this article (10.1186/s12992-017-0310-0) contains supplementary material, which is available to authorized users.

## Background

Health system strengthening (HSS) is a growing area of development assistance for health that aims to support WHO’s six health system building blocks of service delivery, health workforce, supply systems, financing, health information systems and leadership [1]. In 2015, $2.7 billion in development assistance went to HSS, an 80% increase in yearly spending since 2004 [2].

One important HSS funder is Gavi, a public-private alliance that supports new and underused vaccines in developing countries. In 2004, a report and stakeholder meeting determined that weak health systems were undermining Gavi’s immunization program investments [3]. In response, Gavi began offering HSS grants in 2005. HSS support has continued to expand, and in 2015 $170 million of Gavi’s $1.6 billion in grants went to HSS [2]. The objective of Gavi HSS grants is to increase and maintain immunization coverage by overcoming health system bottlenecks that impede progress. While each country chooses to spend Gavi HSS funds differently, most interventions target relatively downstream activities such as service delivery and supply procurement [4]. Some countries have also used HSS funds to improve health management information systems.

As a relatively new form of funding, less is known about the challenges of implementing system-level programs, as compared to knowledge of medical and behavioral interventions at the individual, interpersonal, or community level [5]. To build this body of knowledge and evaluate their funds and programs, Gavi requires HSS grant evaluations [4, 6, 7]. While findings vary by country, common barriers to HSS implementation include poor program management; lack of guidance from Gavi; weak monitoring and reporting systems; lack of clarity about the scope and goals of HSS support; and poor understanding of reprogramming, which is Gavi’s process for course-correcting programs by revising activities or funds [7].

Gavi’s HSS support in Chad and Cameroon, two of the lowest-performing countries in terms of meeting the Millennium Development Goals by 2015, had not been evaluated prior to this study. Out of 188 countries, Chad and Cameroon ranked 175th and 176th for child mortality progress, and 181st and 178th for maternal mortality progress, respectively [8]. Both Cameroon and Chad face significant health system limitations. For instance, the World Health Organization (WHO) recommends 23 doctors, nurses and midwives per 10,000 population, but Chad and Cameroon have only four and six per 10,000, respectively [9]. Recognizing that such systemic conditions inhibit immunization programs, Gavi began providing both countries with HSS support nearly a decade ago. However, quantitative analyses found no evidence that Gavi HSS funding positively impacted immunization coverage in either country. In Chad, coverage changes were small and did not differ across HSS and non-HSS districts [10]. Coverage increased slightly in Cameroon, but at the same rate in HSS-priority and other districts [11]. These null findings raise pressing questions as to why HSS funds did not achieve the desired effects.

The growing field of implementation science offers a set of tools that should be applied to answer such questions and maximize the impact of future HSS programs, especially as Cameroon and Chad prepare for second rounds of HSS funding [12, 13]. One of the most common implementation science frameworks is the Consolidated Framework for Implementation Research (CFIR), which was developed by Damschroder et al. based on a review of published implementation theories and empirical reports [12]. CFIR is specifically designed for assessing complex, multi-level implementation contexts, which makes it well suited for evaluating HSS programs.

This study is the first to evaluate the Gavi HSS implementation process in either Cameroon or Chad. Identifying the drivers of and barriers to implementation will build a useful evidence base to improve the effectiveness of a large and increasingly important stream of development assistance in these priority countries and similarly challenging settings.

## Methods

We triangulated quantitative and qualitative data to retrospectively evaluate the implementation of Gavi’s HSS support in Chad and Cameroon, and conducted a Root Cause Analysis (RCA) guided by the CFIR.

### Financial analysis

We compared the proposed, planned, and actual budget of Gavi HSS funds. We defined proposed budget as the budget included in a country’s original approved Gavi HSS proposal. We defined planned budget as the budget for the upcoming year specified in the Annual Progress Report countries submit to Gavi each year. Each year’s planned budget can vary slightly from the original proposed budget. We defined actual budget as the country’s actual expenditure, as reported for the past year in the Annual Progress Report or by an external audit. Data analyzed included: Chad and Cameroon’s HSS proposals; Cameroon’s 2008, 2009, 2010, 2011, and 2014 Annual Progress Reports; a financial audit report for Cameroon [14]; and Chad’s 2008, 2009, 2010, and 2013 Annual Progress Reports. Source documents are available at www.gavi.org/country/cameroon/documents/ and www.gavi.org/country/chad/documents/.

### Document review

A comprehensive document review was performed to refine research questions, identify stakeholders for interviews, refine topic guides for key informant interviews (KIIs), and collect factual information about the HSS programs’ components and timelines. Documents analyzed included grant proposals, Gavi responses to these proposals, Gavi HSS guidelines and reports, country annual progress reports, Expanded Program on Immunization (EPI) annual plans, Millennium Development Goals progress reports, national health development plans, and past evaluations of Gavi HSS in other countries.

### Field visits

Field visits were conducted to evaluate the extent to which HSS activities had been implemented. We selected the sample to capture variation by location and type of facility in Cameroon, and variation between HSS and non-HSS targeted districts in Chad.

In Cameroon, we first selected regions to represent the country’s linguistic variations (Anglophone vs. Francophone). We then selected districts within these regions that represented Cameroon’s variation in terms of demographics (rural vs. urban) and vaccination coverage (high coverage vs. low coverage). In each district, we randomly selected public Integrated Health Centers (IHC), which are targeted through HSS funds, and private IHCs, which are not. In Chad, we first selected HSS-targets districts that represented the country’s variation in terms of demographics (rural vs. urban) and vaccination coverage (high coverage vs. low coverage). We then selected non-HSS-target districts from the same regions as the selected HSS-target districts, matched based on the same demographic and vaccination coverage criteria. In each HSS-target and non-HHS-target district, we randomly selected health centers. The list of districts visited are shown in Additional file 1: Appendix Table S1.

Questionnaires were developed around checklists of activities proposed in the original and reprogrammed HSS applications. Chad’s questionnaires did not reflect the activities from the second reprogramming, which was only just being implemented during data collection. The questionnaires were tailored to different levels of the health system, including administrative and service-delivery sites at the health systems’ central, regional, district and health center levels. Trained research assistants administered the questionnaires.

### Key informant interviews

In each country, a list of key informants (KI) was developed based on document review, with input from Gavi and the ministries of health (MOH). KIs included stakeholders from the central, regional, and district MOH; partners from non-governmental and bilateral organizations; and Gavi Independent Review Committee (IRC) and Secretariat staff. Topic guides were developed using the results of document review and Gavi’s evaluation domains, and included questions to understand the process, barriers, and drivers of implementing programs supported by Gavi HSS. Topic guides were customized depending on the respondent, and interviewers probed beyond the standard questions as appropriate. Verbal consent was obtained from all participants. Interviews were conducted by senior researchers and trained research assistants in person, expect for four via Skype. Interviews were audio-recorded and transcribed verbatim.

### Analysis

Key informant interview transcripts were analyzed through thematic analysis, using an iterative coding process to identify important features of the data. Codes were initially based on the overarching research questions, but researchers also inductively derived new codes based on the data. We referenced the CFIR constructs to identify, revise, and structure themes and results from these codes [12]. The CFIR is organized into five domains, each of which contains between four and eight constructs, which in turn have up to six sub-constructs each. The “Inner Setting” and “Process” domains were most relevant, as the evaluation focused on understanding and making recommendations related to institution-level barriers during the implementation process. We also identified some factors in the “Outer Setting” domain.

We triangulated data from all sources to conduct an RCA aimed at understanding key HSS implementation challenges [15]. RCA is a structured method to retrospectively analyze adverse outcomes and identify the chain of events and underlying problems that led to the challenge observed. For this analysis, the researcher asks “why” for each identified cause, and continues to do so until the root cause is identified. As a rule of thumb, the researcher will ask “why” a total of 5 times [16]. RCA is used in a variety of contexts, from understanding poor patient outcomes in a clinical setting to analyzing programmatic challenges for complex interventions like HSS. We used the CFIR domains, constructs, and sub-constructs to guide and structure the RCA analysis and reporting.

## Results

### Sample description

In Cameroon, we administered 124 field visit questionnaires, including one to a central-level Ministry of Health (MOH) representative, one to a partnering organization, seven to regional-level MOH delegates who head the health system in each region, ten to district medical officers, and 105 to IHC managers (Table 1). We conducted 43 KIIs, including four with Gavi Secretariat and the IRC, 11 with health partners including WHO, UNICEF and civil society organizations, 11 with central-level MOH representatives including Directors of relevant divisions or departments such as public health, maternal and child health and EPI, seven with regional-level MOH delegates, and ten with district chief medical officers.

**Table 1:** Field visit questionnaires and key informant interview samples

	Field visit questionnaires		Key informant interviews	
Study audience	Cameroon	Chad	Cameroon	Chad
Gavi Secretariat and IRC	–	–	4	5
Health partners^a^	1	–	11	8
MOH, central level^b^	1	5	11	14
MOH, regional level^c^	7	3	7	3
MOH, district level^d^	10	9	10	9
Health center managers	105	40	–	–
Total	124	57	43	39

^a^Including WHO, UNICEF and civil society organizations

^b^Directors of relevant divisions or departments from the Ministries of Health in Cameroon and Chad, such as public health, maternal and child health, EPI

^c^Regional delegates who head the health system in each region of Cameroon and Chad

^d^District medical officers

In Chad, similarly to Cameroon, we administered 57 field visit questionnaires. This included five to central-level MOH representatives, three to regional-level MOH delegates, nine to district chief medical officers and 40 to health center managers (Table 1). We conducted 39 KIIs including five with Gavi Secretariat and IRC representatives, eight with health partners, 14 with central-level MOH representatives, three with regional-level MOH delegates, and nine with district chief medical officers.

### Timeline of key events and expenditures

**Fig. 1:**
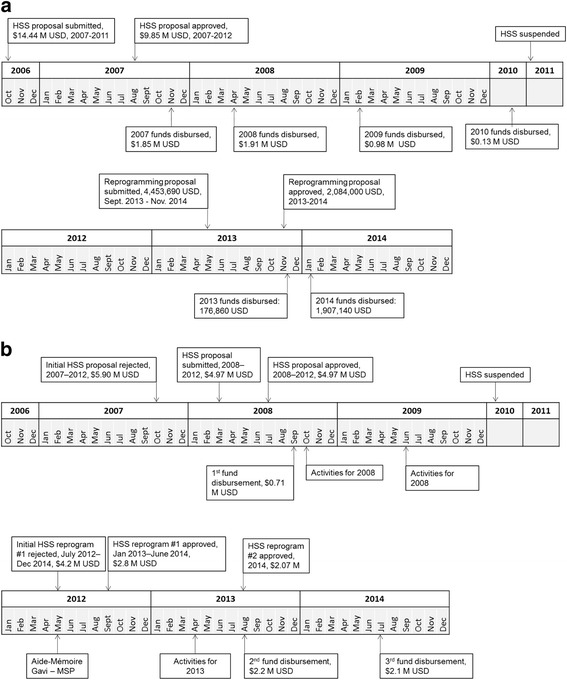
**a**: HSS Milestones, Cameroon, 2006–2014*.* **b**: HSS Milestones, Chad, 2007–2014

Cameroon requested a $14.4 million Gavi HSS grant in October 2006 (Fig. 1a). They were approved for a national, five-year $9.9 million grant in August 2007. Gavi made the first disbursement that November, followed by subsequent disbursements in 2008, 2009, and 2010 (13). Almost 60% of activities were implemented outside the approved budgets. Only $1.9 million of $4.9 million received were spent, far less than the proposed $8.2 million and planned $9.4 million for those years (Tables 2 and 3). HSS funds were spent disproportionately on Integrated Monitoring (3% planned, 14% actual) and Integrated Supervision (15% planned, 25% actual), but only $8000 of $1.9 million were spent for Integrated Coordination. Substantial funds went to purchasing vehicles, reportedly for supervision and management. In response, Gavi conducted an audit and suspended Cameroon’s HSS program in 2011. It was reprogrammed in 2013 for $4.1 million with new leadership, as the prior management was dissolved following the suspension of HSS. Cameroon’s HSS activities were completely changed through the reprogramming, with no relation to activities proposed in the first period. A subsequent disbursement of $3.3 million, ($2.1 million plus some already in-country), was received in early 2014, and Cameroon had spent this amount by the time of data collection (14) (Table 4).

**Table 2:** Cameroon: proposed, planned, disbursed, and actual expenditures of HSS funds before reprogramming, 2007–2011 (1000 s USD)

Category	Proposed	Planned	Disbursed	Actual
Integrated planning	$4237	$5121	$4870	$951
Integrated monitoring	$335	$262		$260
Integrated supervision	$707	$1434		$477
Integrated coordination	$2058	$1858		$8
Support activities	$912	$721		$208
Total	$8249	$9396		$1904

**Table 3:** Chad: proposed, planned, disbursed, and actual expenditures of HSS funds before reprogramming (1000 s USD)

Category	Proposed	Planned	Disbursed	Actual
Organization & management of health services	$1066	$107	$707	$465
Management of drugs & medical products	$720	$268		$149
Program management	$105	$29		$93
Human resources	$51	$210		$0
Total	$1941	$614		$707

**Table 4:** Cameroon: proposed, planned, disbursed, and actual expenditures of the reprogrammed HSS funds, 2014 through June 2015 (1000 s USD)

Category	Proposed	Disbursed	Actual
Increase demand	$257	$2084	$265
Service delivery	$2424		$1962
Governance	$1461		$1103
Total	$4142		$3331

Chad requested a four-year $4.9 million Gavi HSS grant targeting 10 priority districts in March 2008 (Fig. 1b). The proposal was approved that July, and Gavi disbursed $0.71 million in September. (Table 3). Chad spent the full $0.71 million, which was less than initially proposed for that year ($1.9 million), but more than planned ($0.61 million). Almost 40% of activities were implemented outside the approved budget. Chad spent no funds on Human Resources, which comprised over a third of the planned budget, and spent disproportionately on Organization and Management of Health Services (17% planned, 66% actual). Gavi investigated Chad’s HSS fund management, and suspended the program in 2010. It was reprogrammed in September 2012, and a new director appointed due to concerns over the prior director’s fund management. Chad’s originally proposed HSS activities were mainly kept for reprogramming, with some added and others removed (Additional file 1: Appendix Table S2). Reprogrammed funds were not received until August 2013, when a second reprogramming was already taking place as part of broader efforts to situate Gavi activities within the EPI. All $2.2 million in funds from the second reprogramming were received in July 2014. At the time of data collection, nearly a year after implementation was scheduled to end, Chad had spent $0.93 million of $2.2 million proposed for reprogramming (Table 5). Expenditures still differed from what was planned, but less severely than before reprogramming. While not part of the budget analysis or field visit questionnaire analysis, KIs reported that only 10% of funds from Chad’s second reprogramming had been absorbed, despite already passing the scheduled end-date.

**Table 5:** Chad: proposed, planned, disbursed, and actual expenditures of the first reprogrammed HSS funds (1000 s USD)

Category	Proposed	Planned	Disbursed	Actual
Organization & management of health services	$1231	$181	$2200	$54
Management of drugs & medical products	$888	$491		$440
Human resources	$63	<$1		$0
Transfers to districts	$0	$706		$408
HSS II	$0	$23		$32
Total	$2183	$1401		$934

### Partial implementation of planned activities

The field visit checklists captured the implementation status of proposed reprogrammed HSS activities in 2015 in Cameroon and Chad, nearly a year after implementation was scheduled to end. Broadly, implementation was high at the central level, and declined for districts and health centers.

All eight central-level activities planned in Cameroon were complete, except preparing EPI procedure and financial management handbooks (Additional file 1: Appendix Table S3a). All region-level activities were implemented in at least 71% of surveyed regions, except holding quarterly regional coordination meetings (29% of regions). District-level activities were implemented in an average of 58% of districts, including some fully implemented (solar fridge maintenance training) and others absent (phone maintenance) (Table 6). On average, IHC activities were implemented in 61% of public and 33% of private facilities. Public outperformed private on several service-delivery, logistics, and leadership measures.

**Table 6:** Status of HSS implementation in Cameroon at the Integrated Health Center (IHC) level, October 2015

	Goal	IHC public *N* = 48	IHC private *N* = 57
Community engagement	Train 4 community members per health area in routine immunization, search of dropouts, and immunization sessions’ planning	50%	50%
Service delivery	Transport vaccination teams for monthly advanced & mobile strategies	67%	26%
Service delivery	Provide a snack to technical staff providing immunization in advanced & mobile strategies	33%	32%
Service delivery	Provide per diem to community members who conduct social mobilization & mobile strategies	27%	32%
Service delivery	Ensure daily vaccination in fixed sessions in health facilities by adhering to the open vial rule	94%	32%
EPI logistics	Equip 50 health centers with a solar refrigerator	2%	0%
EPI logistics	Acquire & distribute 422 motorcycles to support immunization activities in remote areas	46%	9%
EPI logistics	Train EPI providers in health facilities of the 20 priority districts	94%	46%
Supervision	Hold a micro-planning meeting in all of the district’s health facilities, with all health sector heads & community health chiefs	71%	40%
Leadership & governance	Conduct a monitoring session in at least 60% of the health areas	71%	28%

Chad implemented all planned central and regional-level activities, except performance-based payments (Additional file 1: Appendix Table S3b). On average, district-level activities were implemented in 72% of HSS-designated districts surveyed; similar activities were implemented in 62% of non-HSS districts. Management trainings, operational plan workshops, and performance incentives (which were ultimately removed for the second reprogramming) were the least implemented. Health-center-level activities were implemented in an average of 57% of HSS districts and 55% of non-HSS districts surveyed (Table 7). Poorly implemented areas included cold chains, solar refrigerators, performance incentives, and training on health information systems.

**Table 7:** Status of HSS implementation in Chad at the health center level, July 2015

Area	Goal	Questionnaire question	Health centers, HSS target districts *N* = 31	Health centers, non-HSS target districts *N* = 9
Planning & coordination	Support the organization’s health activities in fixed, advanced & mobile strategies	Does this facility conduct immunization services in the community?	93%	100%
Monitoring & supervision	Reproduce the tools for EPI management & integrated supervision	During the last supervisory visit, did the supervisor use a checklist on which he noted his comments?	87%	100%
EPI data	Train 8 teams DRS & the DS 10 members in Health Information System (HIS) data & software management	Have any staff at this institution been trained on the use of HIS?	51%	44%
Manager capacity for drugs & vaccines	Perform integrated formative supervision every 3 months on managing supplies & medicines	How many times during the past 3 months has this center received a supervision visit from of the district or regional level?	Avg # visits: 3	Avg # visits: 2.5
Cold chain	Provide the health centers with 100 solar refrigerators	Among your refrigerators, how many are solar?	3%	11%
Cold chain	Maintain the cold chain equipment	Does the cold chain receive regular routine maintenance?	19%	11%
Human resources	Reward the best performing health staff member & DRS	Are facility staff rewarded for their performance?	16%	0%
Human resources	Train 118 health workers at all levels in EPI & prenatal care	Has there been an EPI training for the staff?	87%	100%

### Root causes of Gavi HSS implementation challenges in Chad and Cameroon

**Fig. 2:**
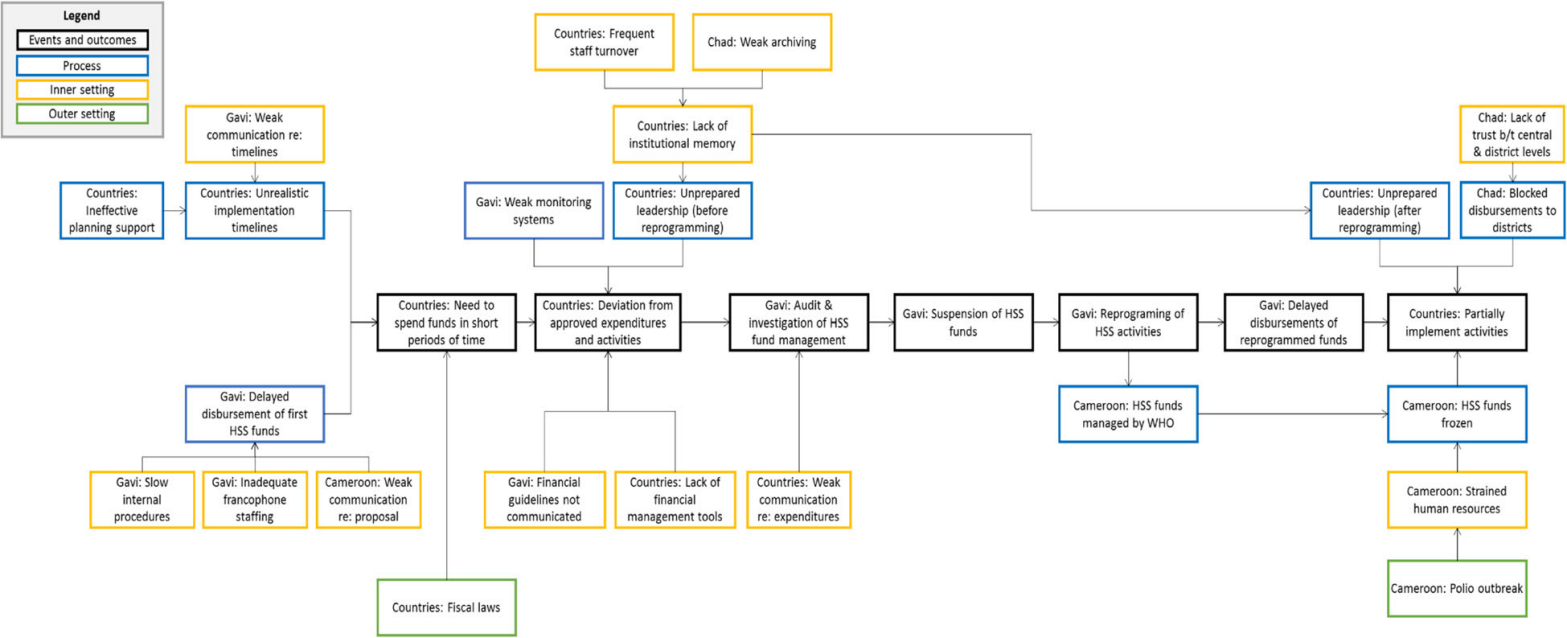
Root Cause Analysis

Figure 2 presents an RCA of the major factors leading to these key events and outcomes, including suspended funds, reprogramming and the partial implementation of HSS activities. We describe below the factors that impeded the implementation of Gavi’s HSS grants in Chad and Cameroon. These factors can be categorized under three domains from CFIR: 1) Process, 2) Inner Setting, and 3) Outer Setting. Each of these is further stratified based on the actor: Gavi and the countries. In general, most events and outcomes are driven by one or more Processes, which in turn are driven by one or more Inner Setting factor. As many themes were shared across Chad and Cameroon, the two countries are presented jointly, with differences noted. The specific CFIR constructs associated with each finding are shown in Additional file 1: Appendix Table S4.

### Processes that impeded HSS implementation at Gavi’s level

#### Delayed disbursement of HSS funds

As detailed above, 10 months passed between submission and approval in Cameroon, and another 3 months before funds were disbursed. In Chad, approval occurred 4 months after submitting the proposal, and the first disbursement arrived 2 months later. KIs from Gavi considered a 10-month delay before approval to be long, whereas two to 3 months to disburse is standard. The countries perceived all these delays as very long. As a result, and in combination with in-country fiscal requirements, they felt forced to spend funds in a short period, often differing from what had been approved. Similar delays were repeated during the reprogramming period.

#### Weak monitoring systems

Gavi’s HSS monitoring system for Chad and Cameroon consisted of a single annual document reporting expenditure and outcome indicators. As described by a KI from Chad, this was an intermittent rather than ongoing process: “this is the annual review…and at the end there is a general report”. This lack of oversight allowed the countries to deviate significantly from the approved expenditures. Without a strong monitoring system to course-correct the countries’ activities, Gavi was left to more reactionary measures including conducting an audit and freezing funds.

### Processes that impeded HSS implementation at the countries’ level

#### Unrealistic implementation timelines

Chad and Cameroon planned to begin implementation a few months after proposal submission. However, disbursements did not arrive until several months after the scheduled start of implementation. The countries did not have contingency plans in place for this situation, leading to rushed spending and deviations from the planned expenditures and activities.

#### Ineffective planning support from technical assistance partners

Chad and Cameroon’s poor planning was unsurprising, given that planning was a known weakness in these countries. In fact, both countries’ HSS plans included measures to improve planning and managerial capacity. Therefore, the unrealistic HSS implementation timelines represent a failure on the part of technical assistance providers, which should have helped the countries anticipate and plan for delays. Both countries repeated similar planning mistakes during reprogramming, again demonstrating a lack of support.

#### Unprepared leadership (before and after reprogramming)

Prior to reprogramming, KIs perceive that the Technical Secretariat leading Gavi HSS in Cameroon was not equipped to manage the funds and programs, largely because it was designed for (and repurposed from) the separate task of piloting a sectoral health strategy. In Chad, the Directorate of Planning changed in between HSS approval and disbursements, due to the appointment of a new Minister of Health, and found that the implementation plan and budget they inherited lacked important details about how the funds should be allocated. Without guided leadership, the programs failed to adhere to the planned activities and expenditures.

After reprogramming, Cameroon and Chad again struggled to secure strong leadership. In Cameroon, a new HSS management team was hired in response to the suspension of funds. However, it took a full 2 months after Cameroon received its already delayed reprogrammed HSS funds to establish this team. In Chad, the prior director was replaced based on the findings of the audit. Unfortunately, the new director was left to manage HSS alone.

#### HSS funds managed by WHO and frozen in Cameroon

During HSS reprogramming in Cameroon, the WHO was tasked with managing their reprogrammed funds to avoid additional delays while the country prepares HSS-specific financial guidelines. According to KIs in Cameroon, this was a natural partnership: “We chose to go to WHO because we had total confidence in WHO...It was not an exception, it was already a practice from our own funds and activities. We went through WHO as there was a shortage of procedures, and WHO already has its procedures.” Indeed, financial analysis shows that WHO ensured compliance with Gavi’s financial management procedures, and Cameroon’s reprogrammed expenditures better aligned with the approved budget (Table 4). However, channeling funds through WHO meant that HSS funds were tied to other aspects of the WHO financial system. Unfortunately, this meant that when the entire WHO financial system was blocked for 4 months in 2013, no HSS activities were implemented during this period, further hindering progress towards Cameroon’s reprogrammed activities.

#### Blocked disbursements to districts in Chad

Chad’s reprogrammed plans called for transferring $0.71 million directly to districts. However, only $0.41 million were transferred, reportedly because transfers were blocked by the central level. This was an important reason for many of the planned activities not being implemented in Chad, particularly at the district level.

### Inner setting factors that impeded HSS implementation at Gavi’s level

#### Slow internal Gavi procedures and inadequate francophone staffing

Gavi’s long approval and disbursement timeline was due in part to inefficient internal financial procedures. There were significant delays related to assessing budgets and financial management plans, which required a hierarchical series of signatures and approvals. As stated by a Gavi KI, the time between submission, approval and disbursement was “extended with the new procedures: financial capacity evaluation, receipt of audit report, consideration of the report and decision following the results of the audit, lack of details provided by the country on the bank account, time between the approval and the disbursement. This period is justified by the search for solution to the problems detected by the audits.” Gavi’s already slow internal procedures were compounded by the lack of francophone staff at the time of Chad and Cameroon’s applications, leading to the long delays in disbursements.

#### Weak communication of realistic timelines

Although previous countries’ HSS application processes had been similarly slow due to Gavi’s internal procedures, Gavi did not communicate realistic timelines for approving and disbursing funds to Chad and Cameroon. This contributed to the countries creating unrealistic programmatic timelines, which were impossible to adhere to given the actual timing of disbursements.

#### Financial guidelines not adequately communicated

While Chad and Cameroon’s implementation timelines for the first round of HSS were unrealistic, Gavi did not offer clear guidelines for how the countries should manage their finances, which led to large deviations from the approved expenditures and activities. A KI in Cameroon reported that financial management guidelines were somewhat improved after reprogramming: “Now, when there were problems with the reprogramming, Gavi gave no directives - but from the agreement from 2013, there were guidelines on the management of the funds.” They also reported a stronger presence from Gavi overall after reprogramming, stating “GAVI has been present at all stages.”

### Inner setting factors that impeded HSS implementation at the countries’ level

#### Weak communication to IRC during proposal process

In addition to Gavi’s internal procedures, delays in Gavi disbursements were also partly attributable to poor communication from the MOH to the Gavi IRC during the application review process. For instance, documents show that Cameroon took several months to respond to IRC queries and clarifications, which KIs report was partly due to high staff turnover and weak telecommunication systems. As a Gavi KI explained, “The big problem with Chad is the internet or even the phone. It is the country that poses more problems to send a report or respond to an email. The communication system is not good, but there is no other options. The problem is technology but people do what they can.” Similar problems occurred during reprogramming, again slowing the disbursement process.

#### Lack of financial management tools

Once the delayed disbursements were received, Chad and Cameroon had few tools to determine how to manage and spend the funds. For example, the Technical Secretariat in Cameroon had no clear management tools and standards from 2008 to 2010, enabling a broad interpretation of what spending was allowed. As one KI stated: “There was some blurring in spending procedures, the Gavi fund expenses were not clear, it was not stolen money, but people were using the money for activities that were not eligible, unknowingly, but have been declared ineligible.” This, coupled with the lack of guidance from Gavi, unprepared leadership, and pressure to spend money quickly, led the countries to deviate from their planned activities and expenditures.

#### Weak communication to Gavi about expenditures

Unfortunately, Chad and Cameroon failed to communicate with the Gavi Secretariat when they began deviating from the approved activities and expenditures. Gavi must approve any budget change exceeding 15%, and both countries far exceeded this. Cameroon KIs reported they did not proactively communicate its significant changes to Gavi. Some KIs from Chad stated that they did notify Gavi, but never received a response: “We made an annual plan for expenses that we sent to Gavi, who did not responded to our request, and hence we perceived the plan as accepted. Once we bought vehicles, [Gavi’s representative] came to say that this is not the priority…Gavi said that vehicles purchased was a defrauded case. Yet it was planned.” Gavi KIs did not acknowledge receipt of notice from Chad. This lack of communication combined with weak monitoring systems led Gavi to investigate, audit, and ultimately suspend the first round of HSS funds in both countries.

#### Lack of institutional memory due to frequent staff turnover and weak archiving

Chad’s repeated experience of new and unprepared leadership reflected a broader culture of frequent staff turnover and weak archiving practices. For instance, our research team frequently found that essential HSS project documents were unavailable for the study. Without institutional memory about the HSS grant, new directors were left to make their own decisions of how to spend funds given short timelines and weak financial guidance, which often did not align with what was originally approved by Gavi. Both countries also demonstrated failure to learn from past mistakes through repeated critical errors including setting unrealistic implementation timelines without contingencies for late or partial disbursements.

#### Lack of trust between central and district levels of health system in Chad

In Chad, one of the biggest deviations from the planned reprogrammed activities were the blocked disbursements from the central MOH to the districts. Underlying this was a culture of distrust between the central and district levels in Chad’s health system. The Minister of Health in Chad, newly appointed at that time, suspended all transfers from the central to the operational level in an attempt to better manage MOH finances.

#### Strained human resources in Cameroon

In Cameroon, the blockage of WHO-managed funds including HSS funds was primarily attributed to the fact that there were not enough administrative staff to process an overwhelming number of receipts from 14 polio campaigns conducted in 2013.

### Outer setting factors that impeded HSS implementation at the countries’ level

#### Inflexible existing fiscal laws

Both countries were subject to fiscal laws that prevented them from rolling funds over to a later period. When the initial funds from Gavi were received later than expected, these laws forced them to spend HSS funds in short periods.

#### Polio outbreak in Cameroon

In Cameroon, the MOH implemented 14 vaccination campaigns to face a large polio outbreak in 2013. These campaigns were financially managed by the WHO whose financial system requires receipts for all expenditures. With the strain on human resources at the MOH, receipts were delayed up to 3 months causing WHO’s financial system to block all transfers, including those of HSS.

## Discussion

This study provides the first insights on the multiplicity of factors hindering implementation of Cameroon’s and Chad’s Gavi HSS programs. Chief among these were unpredictable Gavi processes and disbursements, poor communication between the countries and Gavi, insufficient country planning without adequate technical assistance, lack of country staff and leadership, and weak systems to manage finances and promote learning during implementation. It is notable that many of the weaknesses exhibited by Chad and Cameroon were the same issues their HSS grants intended to address, indicating the need to increase technical, managerial and other forms of non-financial support during HSS implementation [17].

In Gavi’s early years, countries perceived that the application process was too rapid and complex [18]. While this study and prior evaluations find the HSS experience has been slower, it has become more complex, uncertain, and difficult to navigate [7]. Gavi should revise protocols, streamline approvals, and reinforce staff to make application and disbursement processes more predictable and timely. Pooled or joint fund management may be one promising option, having improved processes in Ethiopia, Nepal, and Sudan [19–21]. In tandem, Gavi should strengthen communication frameworks with countries, addressing a common barrier to scaling up programs [22]. Better communication is needed to clarify the application process, hold countries accountable for communicating budget changes, and guide countries on how to proceed when disbursements are delayed or divided. One concrete action would be increasing in-country presence via Senior Country Managers, as recommended by the recently completed Gavi Full Country Evaluations [23, 24]. Communication improvements should also be complemented by codified policies on how to proceed with delayed disbursements. Until Gavi’s disbursements become more predictable, Chad, Cameroon, and other countries would be wise to include contingencies in their proposals such as tying implementation start dates to disbursements, developing smaller alternative plans in the case of late or partial disbursements, and/or identifying alternate donor or government funds to cover activities during delays.

A second theme was the need for countries to improve their learning and knowledge-management practices, especially given the high rate of staff turnover. While Gavi, Chad and Cameroon showed some isolated examples of learning from past mistakes (eg: development of financial management guidelines after reprogramming), many critical problems such as unrealistic timelines and unprepared leadership were repeated multiple times. Looking beyond HSS, Chad also failed to learn from a similar experience several years earlier, when a Global Fund-supported program was suspended due to mismanaged funds [25]. A number of existing frameworks can facilitate productive learning during implementation, many of which are based around the Plan-Do-Study-Act (PDSA) Cycle for quality improvement [26–28]. These frameworks typically advocate a sequential approach that allows for testing and adapting interventions prior to scale up, rather than the single sweeping introductions that occurred in Chad and Cameroon [26]. Some donors have begun to adopt learning frameworks [29]; Gavi specifically has used “learning agendas” to study implementation issues for the cholera and rabies vaccines and commissioned several prospective evaluations [30, 31]. Unfortunately, a learning mindset was not apparent during Chad and Cameroon’s HSS programs, and an intentional approach is needed to ensure the lessons from this current experience are not lost as those from the Global Fund were.

For HSS to succeed, Gavi and countries should prioritize strengthening essential functions necessary for implementation, such as human resources and managerial capacity. The lack of focus on human resources and other “upstream” efforts has been a focus of past Gavi HSS critiques [32, 33], and this was clearly an issue in Chad, which spent none of the over $200 million intended for human resources. Human resource challenges exemplify the circular logic of HSS grants that implicitly expect countries to possess the very capacities they are aiming to improve [34]. Human resources and managerial capacity have long been recognized as key barriers program implementation for Gavi, Cameroon and Chad [18, 35] so it is unsurprising that managerial and health worker training were integral components of these countries’ HSS proposals. However, human resources shortages and weak management severely limit countries’ capacity to absorb funds and preclude implementation of scaled health interventions [36, 37]. This was clearly seen in the lack of leadership exhibited in Chad and Cameroon. To overcome the paradox of expecting success from weak areas, countries need to strengthen certain key capacities – such as training staff expressly for HSS program management – prior to implementing the rest of the program. This could be achieved during an inception phase, or with sustained support from a partnering organization. Several initiatives have shown successful models for supporting countries’ planning and managerial capacity [38], but there is a need to adapt these to the HSS context [39–41]. Sequential implementation and learning approaches may also help identify and develop critical functional capacities necessary for implementation such as management abilities, information technology and communication systems. Further, improving countries’ core implementation capacities in the long-term requires policy dialogue, which the Paris declaration for aid effectiveness designates as an integral part of financial assistance for health [42]. The limited policy-focused activities, and the lack of their implementation in Cameroon and Chad HSS, such as developing financial or managerial guidelines, have weakened the role of the local leadership. This has led to the need for a third party to manage HSS finances in Cameroon, and the decreased potential for sustainable gains and capacity to implement future programs in both countries.

Our study is subject to several limitations. First, the archiving problem made it impossible to access or even verify the existence of some important documents. Second, several KI were not available, especially those who played important roles at the beginning of HSS. Finally, recall bias was a limitation as these programs started over 8 years ago. Nevertheless, our study has two major strengths. First, it is based on a mixed methodology that triangulated data from different sources to provide more robust findings. Second, it was an independent evaluation conducted as a collaboration between interdisciplinary local and international teams to create a collectively deep body of knowledge.

## Conclusions

Though Chad and Cameroon both critically needed support to strengthen their weak health systems, serious challenges drastically limited implementation of their Gavi HSS programs. Implementation of future HSS programs in these and similar settings can be improved by transparent and reliable procedures and communication from Gavi, proposals that account for countries’ programmatic capacity and the potential for delayed disbursements, implementation practices that foster learning and adaptation, and a proactive emphasis on developing managerial and other human resources.

## Additional file

Additional file 1: Appendix Table S1. Districts visited for field visit checklists. **Appendix Table S2.** Changes to Chad’s planned HSS activities, before and after reprogramming. **Appendix Table S3a.** Status of HSS implementation in Cameroon, October 2015. **Appendix Table S3b.** Status of HSS implementation in Chad, July 2015. **Appendix Table S4.** Results from the Root Cause Analysis organized by Consolidated Framework for Implementation Research (CFIR) domains and constructs. (DOCX 27 kb)

## Abbreviations

CFIR: Consolidated Framework for Implementation Research
EPI: Expanded Program on Immunizations
HSS: Health System Strengthening
IRC: Independent Review Committee
KI: Key informant
KII: Key informant interview
MOH: Ministry of Health
RCA: Root cause analysis
WHO: World Health Organization
