# Supplementary material for: Challenges to implementing Gavi’s health system strengthening support in Chad and Cameroon: results from a mixed-methods evaluation
Source: Global Health. 2017 Nov 16;13:83. doi: 10.1186/s12992-017-0310-0 (PMC5691914; doi:10.1186/s12992-017-0310-0)
Supplement: Additional file 1: Appendix Table S1. — Districts visited for field visit checklists. Appendix Table S2. Changes to Chad’s planned HSS activities, before and after reprogramming. Appendix Table S3a. Status of HSS implementation in Cameroon, October 2015. Appendix Table S3b. Status of HSS implementation in Chad, July 2015. Appendix Table S4. Results from the Root Cause Analysis organized by Consolidated Framework for Implementation Research (CFIR) domains and constructs. (DOCX 27 kb) [file 12992_2017_310_MOESM1_ESM.docx]

Additional file 1: Appendix Table S1. Districts visited for field visit checklists

| **Cameroon** |
| --- |
| Ngaoundéré Urbain |
| Ngaoundéré Rural |
| Djoungolo |
| New Bell |
| Bamenda |
| Ndop |
| Malantouen |
| Ambam |
| Lolodorf |
| Limbe |
| Muyuka |
| **Chad** |
| **HSS-targeted Districts** |
| Doba |
| Mao |
| N’Djamena |
| Pala |
| **Control Districts** |
| Bessao |
| Lere |
| Moussoro |

Additional file 1: Appendix Table S2. Changes to Chad’s planned HSS activities, before and after reprogramming

| **Original activity** | **Status after reprogramming** |
| --- | --- |
| **Health districts organization and management** |  |
| Support the organization of micro-planning workshops in the 10 health districts (HD) | Eliminated |
| Organize 2 PAO validation and adoption workshops in the 10 HDs | Modified |
| Support the monitoring meetings for actions in the 10 HDs | Maintained |
| Organize quarterly HSS activities monitoring and control meetings at the central level | Maintained |
| Organize and external audit | Maintained |
| Support the annual PRDS review meetings for actions in 8 Health Delegations | Maintained |
| Organize 3 reviews (twice a year) of the PNDS at the central level | Modified |
| Provide the health centers selected in the 10 Districts with 100 motorcycles for the advanced strategies | Implemented prior to change |
| Acquire 20 motorcycles for HC | Maintained |
| Acquire 2 4x4 vehicles for the 2 HD whose vehicles are not functional (Bébedjia and Sarh) | Maintained |
| Support the organization of health activities in fixed, advanced and mobile strategies, including market days | Eliminated |
| Provide the central EPI with a truck to distribute consumables, tools, cold chains, drugs and EPI consumables | Eliminated |
| Organize quarterly integrated supervision missions at the level of the HDs and DSRs concerned | Maintained |
| Provide the central HSS coordination office and the EPI with two all-terrain supervision vehicles | Eliminated |
| Provide 6 central units, 8 DSRs and 10 DSs with computer and office equipment kits to enter and process the data | Implemented prior to change |
| Provide EPI supervisors in 8 Gavi HR with complete IT kits | Maintained |
| Train the members of the 8 DSR teams and of the 10 Districts in GESIS data and software management | Eliminated |
| Train the health committees in the 10 HDs | Eliminated |
| Identify and train the community health care workers in the Responsibility Zones | Eliminated |
| Organize monthly meetings to monitor the management committees and include the community health workers | Eliminated |
| Put in place communication media such as posters, image boxes, etc., in the 10 HDs | Eliminated |
| **Management of essential drugs, medicinal products, and cold chain** |  |
| Train drug and vaccine management staff locally in the 8 regions and the 10 districts | Eliminated |
| Provide the 8 regions concerned with drug management and EPI tools | Eliminated |
| Carry out integrated formative supervision every 3 months in management tools, drugs, child survival activities | Maintained |
| Acquire generic essential drugs for the 100 HC | Maintained |
| Ensure distribution of vaccines, consumables, communication material, and drugs in 8 HR | Maintained |
| Equip the central EPI and the three sub-national depots with four 40 m3 cold chambers each (Moundou, Abéché and Sarh) | Implemented prior to change |
| Install the EPI equipment that is acquired (cold chambers) | Maintained |
| Provide each of the 8 DSRs and the 10 district departments with one freezer each | Maintained |
| Provide the 8 DSRs and 10 district departments with one refrigerator each | Eliminated |
| Provide the 100 health centers with solar refrigerators | Modified |
| Provide drugs to the 100 HC | Maintained |
| Maintain the cold chain equipment | Maintained |
| **Health care human resource capacity** |  |
| Assign 100 more skilled staff to the 10 priority health districts | Eliminated |
| Collect and publish data on the effective presence of the 100 staff members in the 10 Districts | Eliminated |
| Reward staff members with good performance based on merit | Eliminated |
| Reproduce the EPI management tools and the integrated formative supervision tool | Eliminated |
| Organize two mid-level management training sessions for the District Management Teams in the 10 HD | Maintained |
| Train/retrain 118 health care workers from the different levels in EPI, FDD/IRA and PNC | Eliminated |

Additional file 1: Appendix Table S3a. Status of HSS implementation in Cameroon, October 2015

| Central level | | |
| --- | --- | --- |
| Area | Activities | Complete |
| Community engagement | Produce education materials on EPI (posters, leaflets, images, vaccination schedule) | Yes |
|  | Distribute education materials on EPI (posters, leaflets, images, vaccination schedule) | Yes |
|  | Produce & distribute documentary & advertising messages on vaccination on national radio | Yes |
|  | Produce & distribute documentary & advertising messages on vaccination on national television | Yes |
| Supervision | Organize the supervision of the EPI technical committee trainings in the regions | Yes |
| Leadership & governance | Prepare an EPI procedures handbook & financial management guidelines | No |
|  | Support monitoring of Gavi’s reprogrammed HSS & the management of resources by WHO & MOH based on performance-based financing | Yes |
| EPI data | Install 12 teleconference systems before the end of 2013 (one per regional delegation of health, MinSanté & CTG of EPI) | Yes |
| Regional level | | |
| Area | Activities | % of regions visited  (N=7) |
| Community engagement | Produce education materials on EPI (posters, leaflets, images, vaccination schedule) | 71% |
|  | Distribute education materials on EPI (posters, leaflets, images, vaccination schedule) | 86% |
|  | Sign partnerships agreements with 4 radio community channels per region to disseminate messages about EPI | 71% |
| Supervision | Organize the supervision of the EPI technical committee trainings in the regions | 100% |
| Leadership & governance | Hold quarterly regional coordination meetings | 29% |
|  | Train the EPI 10 accountants in the use of TOMPRO management software | 71% |
|  | Prepare an EPI procedures handbook & financial management guidelines | — |
|  | Organize a semi-annual joint WHO-MinSanté financial audit on the management of financial resources in the regions | 71% |
| EPI data | Implement 4 DQS tools in at least 30% of health districts during surveillance in 2014 | 71% |
| District level | | |
| Area | Activities | % of districts visited  (N=11) |
| Community engagement | Train 4 community members per health area in routine immunization, search of dropouts, and immunization sessions’ planning | 73% |
|  | Educate 5 women associations per health district on EPI | 45% |
|  | Sign partnership agreements with 1 community organization per district to educate populations on EPI | 55% |
| Service delivery | Offer a package of integrated services to remote populations on a quarterly basis | 64% |
|  | Organize intensified immunization activities in districts with the largest number of unvaccinated children | 83% |
| EPI logistics | Procure & distribute 10 vehicles to support supervision in priority districts | 17% |
|  | Train 50 office health chiefs in the preventive maintenance & repair of solar refrigerators | 100% |
| Supervision | Organize biannual integrated supervision in at least 80% of the districts | 55% |
|  | Organize supervision of integrated training every two months in at least 60% of health areas | 45% |
| Leadership & governance | Hold monthly meetings for coordination & integrated data review in all districts | 64% |
|  | Hold a meeting to prepare annual action plans in districts | 82% |
|  | Hold a micro-planning meeting in all of the district’s health facilities, with all health sector heads & community health chiefs | 55% |
|  | Conduct a monitoring session in at least 60% of the health areas | 64% |
| EPI data | Train at least 50% of district management teams on using the data quality self-assessment tool (DQS) | 89% |
|  | Implement 4 DQS tools in at least 30% of health districts during surveillance in 2014 | 44% |
|  | Monthly maintenance of phones in health areas | 0% |

Additional file 1: Appendix Table S3b. Status of HSS implementation in Chad, July 2015

| Central level | | | | | | | |
| --- | --- | --- | --- | --- | --- | --- | --- |
| Area | Activities | | Question | | | Number | |
| Monitoring & supervision | Provide HSS & EPI central coordination office with 2 off-road vehicles for supervision | | How many all-terrain vehicles are there in the HSS central coordination office? | | | 2 vehicles | |
| Cold chain | Equip the central EPI & the three subnational deposits with four cold rooms of 40 m^3^ each (Moundou, Abéché & Sarh districts) | | How many 40 m^3^ cold room are there at the central level of the EPI? | | | 14 rooms | |
|  |  |  | How many 40 m^3^ cold rooms are there at the subnational depots? | | | 11 rooms | |
| Regional level | | | | | | | |
| Area | Activities | | | Question | | | % of regions visited (N=3) |
| Planning & coordination: managerial & technical capabilities | Support the annual review meetings of the annual plan in 8 regional health delegations | | | Is there a plan for regional health development for this regional health delegation? | | | 100% |
| EPI data | Train 8 teams DRS & the DS 10 members in Health Information System (HIS) data & software management | | | Was there a training for regional staff on the collection & analysis of HIS data? | | | 100% |
| Manager capacity for drugs & vaccines | Train 8 DRS on managing medicines & EPI tools | | | Has the DRS EPI Manager received training? | | | 100% |
|  |  |  |  | Has the DRS medicines, vaccines & medical products supply chain manager received training? | | | 100% |
|  | Ensure the distribution within 8 regional health delegations in vaccines, consumables, hardware communication & essential generic medicines | | | Is your DRS regularly supplied with vaccines, essential medicines, & medical products from the central level? | | | 100% |
| Cold chain | Provide each of the 8 regional health delegations & 10 health districts with a freezer each | | | How many freezers are available at the regional level? | | | Mao: 10 freezers  Pala: 2 freezers |
|  | Maintain the cold chain equipment | | | Does the cold chain receive regular routine maintenance? | | | 100% |
| Human resources | Reward the best performing health staff member & DRS | | | Are facility staff rewarded for their performance? | | | 33% |
| District level | | | | | | | |
| Area | Activities | Question | | | % case districts visited (N=4) | | % control districts visited (N=3) |
| Planning & coordination: managerial & technical capabilities | Support the organization of goal-setting workshops in 10 health districts (DS) | Was there a goal-setting workshop in this district? | | | 100% | | 100% |
|  | Organizing 2 workshops for validation & adoption of the annual operational plan in 10 DS | Was there a workshop for validation & adoption of the annual operational plan in this district? | | | 50% | | 33% |
|  | Support the organization’s health activities in fixed, advanced & mobile strategies | Is there a district plan for advanced & mobile strategies? | | | 75% | | 100% |
|  | 2 intermediate management trainings for DS management teams | Did any staff attend mid-level management training? | | | 25% | | 0% |
| EPI data | Provide 6 CPUs, 8 regional health delegations & 10 health districts with hardware to capture & process data | Have there been logistical improvements for data collection (e.g., purchase of computers or computer equipment)? | | | 100% | | 100% |
|  | Train 8 teams DRS & the DS 10 members in Health Information System (HIS) data & software management | Were district-level staff trained on the collection & analysis of HIS data? | | | 75% | | 66% |
| Cold chain | Provide each of the 8 regional health delegations & 10 health districts with a freezer each | How many freezers are available at the district level? | | | Mao: 2  N’Djamena: 2  Doba: 1 | | Bessao: 0  1st: 2  Moussoro: 0 |
|  | Maintain the cold chain equipment | Does the cold chain receive regular routine maintenance? | | | 100% | | 66% |
| Human resources | Reward the best performing health staff member & DRS | Are facility staff rewarded for their performance? | | | 25% | | 0% |
|  | Train 118 health workers at all levels in EPI & prenatal care | Has there been an EPI training for the staff? | | | 100% | | 100% |

Additional file 1: Appendix Table S4. Results from the Root Cause Analysis organized by Consolidated Framework for Implementation Research (CFIR) domains and constructs

| Domain | Actor | Construct | Findings |
| --- | --- | --- | --- |
| Process | Gavi | Executing | Delayed disbursement of HSS funds |
|  |  | Reflecting and evaluating | Weak monitoring systems |
|  | Countries | Planning | Unrealistic implementation timelines |
|  |  | External change agents | Ineffective planning support from technical assistance partners |
|  |  | Formally appointed internal implementation leaders | Unprepared leadership (before and after reprogramming) |
|  |  | External change agents | Cameroon: HSS funds managed by WHO |
|  |  | Executing | Cameroon: HSS funds frozen |
|  |  | Executing | Chad: Blocked disbursements to districts |
| Inner Setting | Gavi | Structural characteristics | Slow internal Gavi procedures |
|  |  | Available resources | Inadequate francophone staffing |
|  |  | Networks and communication | Weak communication of realistic timelines |
|  |  | Networks and communication | Financial guidelines not communicated |
|  | Countries | Networks and communication | Weak communication to IRC during proposal process |
|  |  | Available resources | Lack of financial management tools |
|  |  | Networks and communication | Weak communication re: spending |
|  |  | Access to knowledge and information | Lack of institutional memory |
|  |  | Structural characteristics | Frequent staff turnover |
|  |  | Access to knowledge and information | Chad: Weak archiving |
|  |  | Culture | Chad: Lack of trust between central and district levels |
|  |  | Available resources | Cameroon: Strained human resources |
| Outer setting | Countries | External policy and incentives | Inflexible existing fiscal laws |
|  |  | Does not align with CFIR construct | Cameroon: Polio outbreak |
